# Culturally Safe Nursing: Transformative Learning Experiences Through Critical Reflection Assessment

**DOI:** 10.1002/nop2.70718

**Published:** 2026-08-27

**Authors:** Lesley Andrew, Michelle Pedlow, Bev Ewens

**Affiliations:** ^1^ School of Nursing and Midwifery Edith Cowan University Joondalup Western Australia Australia; ^2^ School of Health and Clinical Sciences University of Western Australia Perth Western Australia Australia; ^3^ School of Nursing Murdoch University Perth Western Australia Australia

**Keywords:** aboriginal health, critical reflection, cultural safety, nursing education, transformative learning

## Abstract

**Aim:**

To explore the transformative learning experiences of postgraduate nursing students engaging in an Aboriginal culture and history module, through an analysis of a critical reflection assessment, in particular the student development of cultural awareness and culturally safe practice.

**Background:**

Despite mandated inclusion of Aboriginal cultural content in Australian nursing curricula, culturally unsafe practices and racism persist. Transformative learning, underpinned by critical reflection, can challenge fixed assumptions and promote cultural awareness and culturally safe care.

**Design:**

A qualitative descriptive study using thematic analysis.

**Methods:**

Written critical reflections from 15 consenting postgraduate nursing students who completed the Ngany Kalyakal Katatjiny Boordawan Module, which was developed to promote culturally safe care of Australian nurses, were analysed using Braun and Clarke's thematic analysis and interpreted through Tsimane and Downing's conceptual model of transformative learning.

**Results:**

The three developed themes were: (1) Students' initial awareness and understanding of Aboriginal culture, history and health—revealing knowledge gaps and societal influences; (2) The learning experience—characterised by emotional responses, identification with Aboriginal experiences and critical re‐examination of traditional healthcare perspectives; and (3) Practice through a new lens—demonstrating conceptual shifts and intentions to apply culturally safe approaches in practice.

**Conclusion:**

Critical reflection of culture and health learning activities can support transformative learning, challenge prior assumptions and facilitate the development of skills and attitudes aligned with culturally safe nursing practice. Careful planning of these learning activities is needed to manage student emotional discomfort.

**Impact:**

Findings highlight the need for authentic, meaningful reflective activities and ongoing evaluation of cultural and health education across nursing curricula.

**Implications for Nursing Practice:**

The findings highlight the importance of integrating authentic, meaningful learning experiences into nursing curricula, including reflective assessment strategies that foster transformative learning and equip future nurses to provide culturally safe care.

## 1 Introduction

In Australia, culturally safe nursing care is critical to mitigate the health inequities faced by Aboriginal and Torres Strait Islander peoples (Australian Institute of Health and Welfare (AIHW) 2023). In recognition, Aboriginal and Torres Strait Islander (from here on respectfully termed ‘Aboriginal’) cultural content has been embedded in all preregistration nursing degrees since 2009 (The Australian Nursing and Midwifery Accreditation Council (ANMAC) 2019). Despite this, culturally unsafe perspectives and practices, including racism, continue to be reported across Australian nursing practice (Best et al. 2025).

To be meaningful, teaching and learning activities that aim to support culturally safe practice with Aboriginal peoples require a transformative education approach. Through this approach, students' established perspectives and underpinning frames of reference on culture, difference and equality are challenged, leading to more open and inclusive behaviours (Blunt 2007). The critical reflection process, in which the student examines the way they think and constructs meaning from this examination, is an intrinsic aspect of transformative learning activities (Savicki and Price 2021).

In this paper the authors analyse postgraduate nursing students' critical reflection assessments to explore their transformative learning experiences of their online engagement in the Ngany Kalyakal Katatjiny Boordawan Module (The Module). This Module was embedded in a Postgraduate Culture and Nursing unit and engaged students in learning Aboriginal history and cultural ways of knowing, being and doing. Students were asked to reflect critically on their experiences of engagement in the Module and to record and submit these reflections in a written assessment piece (Appendix 1). These data were thematically analysed, and findings organised against the Tsimane and Downing (2019) conceptual model of transformative learning in nurse education. Findings are discussed against the principles and aspects of transformative learning in relation to cultural awareness and culturally safe nursing practice. Evidence from this study will inform academic peers considering assessment activities to support the development of culturally aware and safe nurses.

## 2 Background

The study is situated against the backdrop of Aboriginal health inequity and racism in Australia. It explores critical reflection activities, the process of transformative learning and the development of cultural awareness and culturally safe practice. These ideas are now discussed across the contexts of Australian society, higher education and health.

### 2.1 Aboriginal Health Inequity

Aboriginal peoples in Australia experience significantly worse health outcomes than non‐Aboriginal Australians (AIHW 2024a). Data from 2018 reveal the burden of disease among Aboriginal and Torres Strait Islander peoples was 2.3 times greater than non‐Aboriginal Australians. Life expectancy at birth is considerably lower, with 2022 estimates revealing Aboriginal men have a life expectancy 8.8 years shorter, and women 8.1 years shorter than the overall Australian population (AIHW 2025). Major contributory factors to the ongoing and significant health gap include social and cultural determinants of health such as access to services, educational opportunity and social exclusion. The ramifications of British colonisation continue to impact Aboriginal lives today, with trust in and uptake of health services affected (AIHW 2024a). Ongoing prejudice and racism within Australian society further influence Aboriginal people's life experiences, wellbeing, engagement in healthcare services and subsequent health outcomes (Falls and Anderson 2022).

### 2.2 Cultural Safety in Nursing Practice

The recently revised definition of nursing by peak global nursing body the International Council of Nurses (ICN) has captured the evolving geo‐political landscape of health and health care, strengthening the focus on equity and cultural safety for nurses worldwide:

Nursing is a profession dedicated to upholding everyone's right to enjoy the highest attainable standard of health, through a shared commitment to providing collaborative, culturally safe, people‐centred care, and services. Nursing acts and advocates for people's equitable access to health and health care, and safe, sustainable environments (White et al. 2025, 45).

For Aboriginal peoples, a culturally safe healthcare service is crucial to facilitate positive healthcare experiences, interactions, engagement and outcomes. Cultural awareness, the basic understanding of difference and diversity in cultures within and across populations (Hart et al. 2019) is an ‘invaluable entry point’ towards cultural safety (Kerrigan et al. 2020). The achievement of a culturally safe environment is more complex and requires a critical interpretation of the influence of power on health outcomes, including the examination of ethnocentric ideas of western superiority and the interrelated influences of politics, and privilege (McGough et al. 2022). The idea that culturally safe nurses can create a culturally safe healthcare alone is simplistic, as this requires a systemic shift in leadership, policy and power relationships. However, a nursing workforce that understands the concept and importance of culturally safe practice and possesses the skills, attributes and perspectives that align with it, is an important step towards this outcome (Curtis et al. 2019).

### 2.3 Racism and Lack of Cultural Awareness as Challenges to Cultural Safety in Australian Healthcare

Racism occurs when an individual or group applies power to oppress, discriminate or restrict the life opportunities of others due to their race or ethnic origin (Australian Human Rights Commission 2015). Racism is a recognised barrier to the achievement of cultural safety in healthcare (Kairuz et al. 2021). As a key social determinant of health, racism is a considerable detriment to Aboriginal health outcomes, thereby contributing to the inequitable health gap (Markwick et al. 2019). Racism can occur at multiple levels across society. At the institutional level it is defined as ‘the collective failure of an organisation to provide an appropriate and professional service to people because of their colour, culture or ethnic origin’ (Tendler 1999, 3). Within healthcare institutions, the eradication of racism is necessary to achieve culturally safe services.

Racism can also be interpersonal (existing within interactions between individuals) and include abuse, harassment, stereotypical assumptions and the exclusion of others from life opportunities (Australian Human Rights Commission 2015). Interpersonal racism against Aboriginal peoples can be unintentional and subtle, resulting from the misguided belief that all people should be treated equally (the same), rather than equitably (according to need) and from a general lack of awareness of cultural differences and the goals of social justice (Falls and Anderson 2022). Racism in healthcare reduces the accessibility and acceptability of healthcare services for Aboriginal peoples, which negatively impacts health outcomes. More specifically, experiences of racism are directly associated with psychological distress (Temple et al. 2020), which, considering the 2015–2019 age standardised suicide rate for Aboriginal peoples was twice the rate for non‐Aboriginal Australians, is of particular concern (AIHW 2025). The necessary transformative change in healthcare workers' attitudes and systems approaches requires acknowledgement and recognition of racist practices (Australian Government 2022). In raising health professionals' understanding of diverse cultural needs, opportunities and practices, cultural awareness offers a way to mitigate unintentional racist practices.

### 2.4 Nurse Education's Commitment to Cultural Safety Including Antiracist Practice

The Australian Government is committed to close the inequitable gap in Aboriginal peoples' health outcomes (Coalition of Peaks 2020). As the largest healthcare profession in Australia, nurses play a significant role in achieving this (Deravin et al. 2018). The Nursing and Midwifery Board of Australia (NMBA) Code of Conduct for Nurses (NMBA 2018) requires registered nurses to deliver holistic, culturally safe care, whilst advocating for Aboriginal peoples. To prepare Australia's registered nurses for this role, ANMAC (2019) stipulates all undergraduate nursing degrees that lead to eligibility to practice in Australia include a discreet assessed unit of learning on Aboriginal health, history and culture, with content relevant to Aboriginal health outcomes further embedded across the curriculum.

Despite these directives and the subsequent inclusion of Aboriginal content in the undergraduate nursing curricula, racist and discriminatory healthcare practices and environments continue to be documented across Australian healthcare (Health Performance Council, South Australia 2020). Best et al. (2025) for example, report racism against Aboriginal nurses by non‐Aboriginal nurses. These and other reports have recently prompted the NMBA and the Australian Health Practitioner Regulation Agency to release a joint statement regarding racism and discrimination, reiterating their codes and standards and reinforcing their intolerance of this behaviour in practice (NMBA 2025; Australian Health Practitioner Regulation Agency and National Boards 2020).

## 3 Conceptual Framework

### 3.1 Transformative Learning

Mezirow (2000), a seminal writer on transformative learning and critical reflection, explains how, on reaching adulthood, an individual has developed a frame of reference through which they perceive, experience and interact within the world. This can remain fixed unless the individual engages in certain transformative experiences and activities that challenge this frame of reference (Mezirow 2000). Transformative learning is the process through which these challenges are made, resulting in a shift or opening of personal assumptions, beliefs and habits to ones that are more inclusive and malleable to unfamiliar situations (Mezirow 2000). This process includes critical reflection involving the primordial questioning and reconstruction of thinking and behaviour. In terms of culturally safe practice, transformative learning challenges the student's preconceived frame of reference (Blunt 2007; Tsimane and Downing 2019). As Mezirow explains, ‘by far the most significant learning experiences in adulthood involve critical [reflection]—reassessing the way we have posed problems and reassessing our own orientation to perceiving, knowing, believing, feeling and acting’ (1990, 13). Reflection itself requires the individual to think about the way they think, constructing meaning from learning rather than regurgitating facts. It is contextual and descriptive, integrating emotion, behaviour and cognition, leading to shifted perspectives. In contrast, rote learning of ‘facts’ result in shallower learning that limits the desired outcome of the learning process (Savicki and Price 2021).

### 3.2 Applying a Transformative Educational Approach to Promote Culturally Safe Nurses

In nursing education, reflective practice is commonly applied to support learning and is an important facilitator of understanding difficult concepts and health situations, as well as professional self‐awareness, competence and self‐monitoring behaviour, and also an understanding of their role as a social justice agent (Tsimane and Downing 2019). Further benefits include increased capacity for compassion, empathy and an openness to exploring and adopting new ideas among nursing students (Yang and Zou 2025). The process has been found to be successful in helping nursing students become aware of unconscious biases and provide culturally competent care and student perceived confidence to work with Aboriginal peoples (Hunt et al. 2015).

### 3.3 A Conceptual Model of Transformative Learning in Nursing Education

Tsimane and Downing (2019) have completed a concept analysis of transformative learning in nursing, from which they developed a three‐phase conceptual model to clarify and guide the transformative learning process in nursing. This model is applied to our study across data collection, analysis and interpretation.

#### 3.3.1 Phase One of the Model‐ Antecedents Including the Ngany Kalyakal Katatjiny Boordawan Module

The first phase of the model, ‘Antecedents’ concerns the events and skills required of a student on which their transformative learning experience is based. These antecedents include knowledge, understanding, analysis of situations, events, experiences and ability to adapt to presenting challenges. It is reasonable to expect many of the graduate nurses in our study to have prior knowledge and understanding of Aboriginal culture and health, primed by undergraduate education and by nursing practice. Our curriculum review process, however, revealed significant gaps in postgraduate nursing student awareness. This finding led to the inclusion of the Ngany Kalyakal Katitjiny Boordawan Module in the Culture and Nursing unit. Developed by Edith Cowan University (ECU), The Ngany Kalyakal Katatjiny Boordawan Module: ‘I continue to learn’, was co‐designed in partnership with Aboriginal people, including an appropriate Aboriginal leader with expertise in the local Aboriginal Noongar language, history and culture. This partnership, and the inclusion of Aboriginal perspectives and stories, contrasts with the more common approach adopted by Western universities—the lecture offered by non‐Aboriginal teachers without lived experience of the phenomenon (Burgess et al. 2022). The Module included a cultural competency package named ‘Yuwahn‐Wupin,’ which means ‘culturally able’ in Yugambeh language. This package was designed by Griffith University, the Congress of Aboriginal and Torres Strait Islander Nurses and Midwives (CATSINaM (2017)).

The Ngany Kalyakal Katatjiny Boordawan Module applies an evidence‐based approach that includes learning activities on equity, cultural safety, Aboriginal culture, identity and health, social determinants of health and the impact of colonisation on Aboriginal and Torres Strait Islander peoples and their health. The 3‐h Module, which includes interactive quizzes, story videos and readings, is delivered online. The inclusion of this Module enhances the students' antecedent knowledge and experiences on which to base their transformative learning and associated culturally safe practice within the Australian healthcare context.

#### 3.3.2 Phase Two of the Model. The Processes of Critical Reflection and Transformative Learning

Phase two of the model ‘Process/Procedure’ describes ‘A highly cognitive and effective deep structural mental shift that involves a meaningful interactive, integrative and democratic construction process to arrive at new insight and changed perspectives’ (Tsimane and Downing 2019, 93).

This transformative learning process depends on reflective discourse, in which the student is guided to examine their own and other experiences and perspectives to improve their awareness of previously fixed judgements and assumptions (Mezirow 1990; Tsimane and Downing 2019). The stages of critical reflection that prompt transformative change start with an event or events that trigger questioning of prior assumptions (frame of reference). Critical reflection on and/or during an event or experience is usually a solitary process where they explore different perspectives and critically test assumptions and fixed ideas (Morgan and Cieminski 2023). For our students, this included reflection on their experiences of Module engagement.

The critical reflection assessment provided structure to guide student reflection activities. Students were asked to critically reflect on the feelings evoked as they undertook the Module, including if their engagement in the Module had challenged their personal values and attitudes. Students were also asked to reflect on the Module experience with reference to some of the main concepts introduced in the Culture and Nursing Unit, such as equity, social determinants of health and generational trauma. Students were advised to take contemporaneous notes during their engagement in the Module to support their reflection. The students were familiar with the Gibbs seminal model of reflection (Gibbs 2013) chosen for the guiding reflective framework.

#### 3.3.3 Phase Three of the Model—Outcomes. Applying Transformative Learning to Practice

This final phase of the transformative learning process refers to desired outcomes of the transformative process. Outcomes are not restricted to knowledge but include behavioural, attitudinal and emotional domains such as autonomous thinking and widened perspectives. This phase aligns with the assessment requirements that asked students to discuss how their new learning may support their future practice with Aboriginal peoples.

### 3.4 Study Aim

This project aimed to analyse critical assessment pieces to explore the learning experiences of participants who undertook the Ngany Kalyakal Kaadidjiny Boordawan Module.

More specifically, the objectives were to understand:

- The process of transformative learning among nursing students (including shifts in cultural awareness and development of cultural safety attributes)
- The way the students perceived their learning may influence their future professional practice with Aboriginal peoples.

## 4 Method

### 4.1 Design

A qualitative descriptive approach was applied to explore student perspectives and experiences. Data were the written text reflections of students who completed the Module.

The Consolidated Criteria for Reporting Qualitative Research Checklist (COREQ) (Tong et al. 2007) was applied to support the research rigour (Appendix 2).

### 4.2 Sample and Participants

The study was approved by the Edith Cowan University Research Ethics Committee (023–04524‐EWENS). After the Culture and Nursing unit was completed in 2024, students were emailed with participant information letters outlining the purpose of our study, requesting permission to use their assessment work in our research analysis. A convenience sample was used of students who had completed the culture and health unit. Only assessments from students who provided written consent via return email had their assessments analysed. No names or other identifying information was included in the data analysis.

### 4.3 Data Collection and Analysis

The research team consisted of two academics, both of whom had taught the Culture and Health unit and embedded Module, on which the assessment was set. At the time of the study, the unit was taught by a separate academic who was not part of the research team. The research team analysed the text content of the reflection assignments from consenting students. The analysis of this qualitative data followed Braun and Clarke' (2021) reflexive thematic analysis guidelines. This provides flexibility to interpret qualitative data through the Transformative Learning Theory lens, and map participants' progress towards cultural safety. The method was used to inductively identify, analyse and report patterns (themes) within data that respond to the research question and sub questions. The full deidentified assessment texts were read and reread by both authors in an iterative, collaborative data analysis process. Segments of data were manually coded. After revisiting and rereading the text, these codes were developed as sub themes and themes (Table 2). These themes and their subthemes were then interpreted under the three phases of the Transformative Learning Model. The two researchers acted collaboratively across the analysis process, applying a questioning and consensus approach across all stages of coding, thematic development and interpretation. This approach was adopted to demonstrate how students experienced transformative learning; how their awareness and understanding evolved, how they critically reflected on their previous practice and experiences, and how this process is expected to shape their future professional practice.

### 4.4 Trustworthiness

The close application of the COREQ (see Appendix 2) supported the study's trustworthiness across all criteria of the Guba and Lincoln (1989 framework) throughout the design, implementation and data analysis. Credibility was strengthened through the inclusion and examination of theoretical perspectives against findings of the study and a transparent and comprehensive discussion of study limitations in this manuscript. Dependability is supported by the transparent research process, design and context description, including the assessment brief (Appendix 1). Confirmability is enhanced through the inclusion of raw data to illustrate themes and the researcher' reflexive statement (see Discussion). Transferability is supported through the inclusion of participant demographics and detailed definition and discussion of phenomena under study, Finally, the authenticity of the study is demonstrated through the sharing of unaltered and varying student perspectives and experiences through direct quotations of their written reflections, and through the publication of the study findings.

## 5 Findings

Table 1 outlines the participant demographics. All 15 participants were domestic students; however, five of the 15 participants were migrants (not born in Australia). The majority of participants were female (13/15), aligning with the proportion of females to males in the nursing workforce (AIHW 2024b). All were mature‐age students, the majority aged 45 years or over. Participant demographic data on students' identity as ‘white,’ black, etc. was not gathered, however this information is included in findings where the participant self‐identified as such in their written reflections, and where this information has relevance to the findings.

**TABLE 1:** Participant demographics.

Age range in years (n)	Sex	Students' degree subject	Country of Origin
25–29 (1) 30–34 (1) 35–39 (1) 40–44 (4) 45–49 (1) 50–54 (5) 55–59 (1) 60–64 (1)	Female (13) Male (2)	Master of Nursing (Nurse Practitioner) (14) Master of Nursing‐ Graduate Entry (1)	Australia (10) Zimbabwe (2) India (2) Kenya (1)

All but one were undertaking the Master of Nursing (Nurse Practitioner) degree. These students had completed an undergraduate degree and had practiced as a nurse in Australia for at least 5 years prior to beginning the master's level course. The Master of Nursing (Graduate Entry) student had previously completed a bachelor's degree in a non‐nursing subject.

Around a third of students (15/46) who completed the Module gave written consent to the analysis of their assessed critical reflection. Two thirds did not reply to the recruitment post. This may have been due to the fact recruitment took place at the end of a teaching semester when students were taking a break from study with the university.

Table 2 outlines the three overarching themes and their corresponding subthemes, as well as where these themes best align with the transformative learning process.

**TABLE 2:** Theme details.

Theme	Sub themes	Transformative learning model phase
**Students' initial awareness and understanding of Aboriginal culture, history and health**	*Initial cultural awareness*	ANTECEDENTS. Initial knowledge, understanding, perspectives and assumptions of Aboriginal culture, history and health awareness (and associated influences).
	*Identifying family and societal influences on cultural awareness*	
**The learning experience**	*Feelings of anger, distress, shame and guilt*	PROCESS. The process of making sense of feelings evoked during engagement with the Module. Identifying with the experiences of Aboriginal peoples. The development of a critical approach to traditional health care systems and the critical reexamination of practice.
	*Identifying with Aboriginal experiences*	
	*Critical re‐examination of traditional approaches to health*	
	*Critical re‐examination of past practice*	
**Practice through a new lens**	*A new lens of understanding*	OUTCOMES. A new conceptual understanding of Aboriginal health, and the systemic influences. Practical ways to apply this understanding to future nursing practice, including approaches to communication, collaboration and advocacy.
	*Practice through a new lens*	

### 5.1 Themes

Three themes were developed. The first reports the students' initial awareness and understanding of Aboriginal culture and associated influences. Theme two describes their critical reflection of previous practice, identification with the Aboriginal experiences shared in the Module, and their emotional response to this reflective process. The final theme describes participants' reports of an altered conceptual understanding of Aboriginal health and how this ‘new lens of understanding’ would influence their future nursing practice.

#### 5.1.1 Theme 1: Students Initial Awareness and Understanding of Aboriginal Culture, History and Health

This theme considers the students' initial awareness of Aboriginal culture, history and health (prior to their engagement with the Module) and the influences of family, education and society on this awareness.

##### 5.1.1.1 Initial Cultural Awareness

The findings revealed insufficient cultural awareness around Aboriginal culture and history. This phenomenon was noted not only among migrant nurses who were born and may have completed much of their education overseas but also domestically educated nurses who had also practiced in the Australian workforce. Participants discussed a general lack of understanding of the impact of colonisation and culture on Aboriginal health outcomes. This included cultural practices that can impact engagement in health services:

My previous understanding of the health and wellbeing of Aboriginals was suboptimal. I realise my own world view at times lacks a meaningful understanding of how colonisation has shaped current social and health contexts for ATSI (sic). (Australian, NPS)

Myths and misconceptions around Aboriginal life were also evident:

I was surprised that a majority (81%) of the First Nations people lived in the city, as I thought the Aboriginal people loved the bush and avoided the cities, this was my misconception. (Migrant, NPS)

##### 5.1.1.2 Identifying Family and Societal Influences on Cultural Awareness

Some students discussed how their ‘whiteness’ including their white Australian heritage had influenced these prior beliefs and perspectives of Aboriginal peoples:

I have biases of Indigenous people stemming from my white privilege upbringing … in which Black people and behaviours are portrayed in a negative light. (Australian, NPS)

A few students described the inadequacy of school discussions on Aboriginal history:

I had realised how naive I had been, my learned preconceptions have reduced significantly over my life span to date from what I had been initially taught from schooling, societal norms and my parents who were taught by their parents. (Australian, NPS)

A participant who identified as a sixth generation white Australian described how she had come to realise her family had not understood the ramifications of the ensuing power imbalance for the Aboriginal peoples from the loss of land, culture and way of life. (Australian, NPS)

My ancestors lived and worked with Aboriginal peoples. They took land from Aboriginals in the 1830s… as their physical needs were met (work, food, shelter) on the cattle station this was regarded to be enough. They were looked after but never empowered to make their own decisions to advance themselves. (Australian, NPS)

Migrant participants also reflected on their childhood and the influence of social and family norms at the time in Australia:

When I was a child, disrespectful names for First Nations Australians were common and it was rare to have anyone question the use of these words or the impact they may have. (Migrant, NPS)

Participants reflected on their prior lack of understanding of certain behaviours, which was influenced by their own education, which was dominated by the Western model of health and its incongruence with the Aboriginal perspective:

My nursing care was influenced mostly by the Biomedical Model of Health, which emphasises that health is the absence of illness and that being health is attributed purely to individual choices. This being the opposite of the First Peoples' Model of Health. (Migrant, NPS)

#### 5.1.2 Theme 2: Learning Experience

This theme describes the students' descriptions of aspects of the process of transformative learning, including re‐examining and making sense of previously held perspectives and identifying with aspects of Aboriginal experiences. It begins with the emotions and feelings that arose during the engagement with the Module.

##### 5.1.2.1 Feelings of Anger, Distress, Shame and Guilt

Students almost universally recalled negative feelings while engaging with the Module. Terms like ‘discomfort’ and ‘confronted’ were used, and many described experiences of anger and outrage towards the treatment of Aboriginal peoples from colonisation onwards. One student described how she had stopped watching what she referred to as ‘stories of abuse’ because of the distress they caused her. Others talked about the need to seek support to manage their feelings:

On completion, I reflected and discussed with my family to process the weight of this information. (Australian, Master of Nursing Graduate Entry)

Australian students who completed the Module often described feeling a sense of shame associated with their growing recognition of privilege, as well as their limited past knowledge of the experiences of Aboriginal peoples. Complex feelings were evoked in students who identified as coming from a white colonist background. These students commonly described feeling confronted by the material and activities that challenged their prior beliefs and perspectives:

I feel a sense of guilt and shame, that as a white person I have special privileges just because of the colour of my skin. I also feel a sense of personal accountability and disappointment for not consistently recognising how past trauma experienced by Aboriginal and Torres Strait Islander peoples continues to impact their lives today. (Australian, NPS)

Feelings of shame also extended to a migrant student:

I felt ashamed that I knew so little about the complexities of Australian history and had only been taught one story. (Migrant, NPS)

One student described the development of dissonance as a result of the confronting feelings she experienced during the process:

This Module elicited feelings of intense guilt and shame, thus creating confusion about what my role is. The content challenged my core values, including fairness and compassion, leaving me feeling emotional and conflicted. (Australian, Master of Nursing Graduate Entry)

##### 5.1.2.2 Identifying With Aboriginal Experiences

Students commonly described how they had identified and empathised with the experiences of Aboriginal peoples. Students with parental responsibilities, for example, described the stories of the stolen generation as particularly upsetting:

Perhaps this (forced removal of children) resonates deeply with me as I have two young children of my own. (Australian, NPS)

A shared experience of colonisation was a common discussion point in migrant student reflections:

The assimilation policy which led to the stolen generation was more upsetting to me as it reminded me of how similar assimilation policies in my country of birth continue to impact our lives and identities, with the example of children's names being imposed on parents as Christian names. (Migrant, NPS)

Some migrants drew parallels between their own and Aboriginal history:

I feel sad and can empathise with the First Nations people, as India was colonised by the British, I recognise the changes left wrought on our families, robbed of our wealth and men, and treated as slaves. (Migrant, NPS)

A migrant student from Asia drew parallels between aspects of Aboriginal and their own culture:

There were similarities with certain events—new births and family deaths brought families together, it was like an expectation to honour family and their passing souls to heaven. (Migrant, NPS)

All five participants from migrant backgrounds (Africa, India) described personal incidences of racism in Australia:

I became empathetic when I saw the stark differences that Aboriginal and Torres Islander people had to live with on a daily basis and how these differences influenced the availability of healthcare. After arriving in Australia as a foreigner, I discovered that white foreigners and black foreigners are not treated equally. I am of African descent. (Migrant, NPS)

Since I have experienced racism and discrimination in the past, I can relate to the feelings and readily feel empathy for the Aboriginal and Torres Islander community. Because I was born into parents who were formerly part of a British colony, I also understand the significance of and the suffering that results from being ignored, colonised, and losing everything under the control of the coloniser. (Migrant, NPS)

##### 5.1.2.3 Critical Reexamination of Traditional Healthcare and Education Perspectives

Students' reexamination of the biomedical model they had been trained in included discussions of its inadequacy as a way of understanding Aboriginal health needs and ways to address them. Students described a renewed appreciation of the social model of health and with this, the importance of the social determinants on health outcomes:

I now understand how social determinant factors, such as education, employment, early childhood development, and housing impact health outcomes for this demographic. (Australian, NPS)

Students also demonstrated a wider critical understanding of the political and other systemic factors that hold power over Aboriginal health and wellbeing outcomes:

I am mindful that the effects of colonisation in Australia impacts the power dynamic between First Australians' and any dominant institution, like the government, where economic, funding and/or political power is predominantly held by the latter. (Australian, NPS)

I can say the Module has reinforced my understanding and knowledge, and learning more about the National Congress of Australia's First People's gives me hope that self‐determination is a real possibility for the Aboriginal and Torres Strait Islander people. I fear, however, unless the congress is protected from changes in government and budget cycles, consistency in delivery of equitable health care will be compromised. (Australian, NPS)

Students also considered how traditional approaches that ignore cultural difference are unhelpful and insufficient to enact positive change:

Using an ethnocentric approach does not work. Using Western ideas alone to address Aboriginal health and social issues will not result in change. (Australian, NPS)

##### 5.1.2.4 Critical Re‐Examination of Past Behaviour and Practice

With raised awareness, participants reflected on past practices they had witnessed in the healthcare environment. This included incidents when hospital staff had become upset when large numbers of Aboriginal family would visit a patient and when Aboriginal patients had become angry and shout when encountering culturally unsafe practice. One student described how her lack of cultural awareness had left her confused about Aboriginal patients' prioritisation of needs:

I used to not understand why, as simple as it is, that patients choose to risk their health outcome by missing dialysis appointments to attend funerals. However, now that I understand the First Peoples' Model of health, it is of sense that they attend the funerals because their health does not only include the absence of their illnesses but the health of the community. (Migrant, NPS)

Many students also reflected on the incongruence between their own practice and Aboriginal needs:

Overall, this experience challenged me to uncover underlying attitudes, beliefs, and assumptions regarding cultural identity and my role as an unconscious participant (in unhelpful practice). (Australian, Master of Nursing Graduate Entry)

Again, participants described discomfort at this new awareness:

I felt uncomfortable remembering all of the times I moved on quickly to the consultation worried about time constraints and unaware of how closed off my client became. I was reminded of all of the social and cultural determinants of health that could be impacting on my client's health and wellbeing and had not taken these elements into consideration when doing a health assessment. (Australian, NPS)

One student recounted a specific example of practices that ignore cultural needs:

This has also led me to reflect on one of my recent experiences caring for an Aboriginal Elder in the Pilbara region of Western Australia, this encounter reinforced for me the vital role of cultural consideration in healthcare settings. This elderly gentleman was at the end of his life and receiving terminal care in a nursing home, he took longer than expected to pass away, and I couldn't help but wonder if it would have made a difference if he were able to be moved home on to Country, surrounded by those very important connections. (Australian, NPS)

#### 5.1.3 Phase 3. Outcome—Practice Through a New Lens

This theme reports on the students' conceptual shift in understanding of Aboriginal culture and health as well as the practical steps and approaches the participants intend to employ to promote culturally safe practice.

##### 5.1.3.1 A New Lens of Understanding

The participants reported how the Module had supported a shift in perspective that would inform their ongoing practice:

This online Module gave me a new lens through which I see and deliver healthcare to First Australians. (Australian, NPS)

These included the importance of a culturally aware and informed, needs‐led service, equity‐driven care and a positive strengths‐based perspective.

While participants recognised the importance of tailored healthcare according to cultural and social needs, they also recognised the need to avoid stereotyping and the treatment of all Aboriginal people the same:

This requires a balance between cultural sensitivity and recognising each patient as a unique individual, rather than homogenising their healthcare experience based on stereotype. (Australian, NPS)

Some students described difference as positive, to be celebrated. One wrote about how they marvelled at the way the Aboriginal culture *‘*has endured, is spiritually connected, and protective of the land’ (Australian, NPS). Another discussed feeling inspired by the design of the Aboriginal flag and by the resilience of the Aboriginal peoples. (Migrant, NPS).

One participant saw the value of applying Aboriginal perspectives to enrich their own life:

I listened to people talk about their connection to country and how this leads to a sense of calm, a renewing of energy and contributes to their positive health and wellbeing. I felt I could learn from this frame of mind that health was more than just physical and apply it to my life and my clinical practice. (Australian, NPS)

##### 5.1.3.2 Applying New Learning to Practice

Participants described how they would apply their enhanced conceptual understanding of culturally safe care and associated skill development to enhance their practice.

As I progressed through the Module, I documented and reflected on the emotions I experienced and contemplated how to integrate the knowledge gained from the Module into my professional practice. This included conversations with my partner, who is also a healthcare professional, about our roles in closing the gap in effort to promote equity in healthcare. (Australian, NPS)

Dominant areas for discussion were the concepts of culturally appropriate communication and collaboration and the advocacy role in culturally safe practice:

The Module presented crucial information that I was not previously educated on. It was a valuable reminder of the significance of effective communication skills such as using simple language, active listening, being aware of non‐verbal cues, and understanding kinship dynamics. (Domestic, NPS)

I began to consider my current practice and contemplated if I took enough time to build rapport with my First Nations clients and allow them to tell their stories before I moved on to health‐related questions during my assessment. (Australian NPS)

Participants discussed a renewed understanding of the importance of working with Aboriginal communities to find answers to ongoing health issues. Some described the way this could be translated into practice:

I will network with various government structures like the National Aboriginal Community Controlled Health Organisation (NACCHO), The Congress of Aboriginal and Torres Strait Islanders/Nurses and Midwives, and the Aboriginal liaison officer. (Migrant, NPS)

Being an active participant in the smoking ceremonies helps clear our spirits, to welcome all cultures into our organisation and workplaces. (Migrant, NPS)

Students commonly discussed how their raised cultural awareness had improved their confidence in collaborating with and meeting the needs of Aboriginal peoples:

A positive that I have taken from these Modules is that I can interact with First Australians with more confidence due to the knowledge gained from these Modules. (Australian, NPS)

Students discussed a range of practical approaches to enhance the cultural safety of their primary health care practice with Aboriginal communities and individuals. These included the importance of yarning and taking time in health interviews and other interactions. Participants also discussed how they could apply authentic health education messages through art and flexible approaches to health information delivery formats. Participants also described a growing sense of responsibility to enact change. They recognised their role as a nurse in advocating for change for Aboriginal peoples they cared for and worked with:

My role as a nurse is in closing the gap, promoting equity and advocating. (Australian, NPS)

I hold responsibility to practice culturally safe, non‐prejudicial and respectful nursing care to all individuals. (Australian, NPS).

Most students referred to their nursing codes and standards to underpin their ideas of advocacy and responsibility:

The Code of Ethics and the Registered Nurse Standards (Nursing and Midwifery Board, 2017) both outline that nurses need to be able to deliver safe, effective, quality care, which is culturally appropriate, person centred and without prejudice. (Australian, NPS)

Students also identified resources to support ongoing professional development in their advocacy role:

(I will) attend culturally appropriate workshops to start engaging as an advocate for social justice in my local community. The Noongar Boodjar Language Cultural Aboriginal Corporation (NBLCAC) provides a range of cultural competency training workshops. (Australian, NPS)

## 6 Discussion

### 6.1 Researchers' Reflexive Statement

Researcher reflexivity is central to maintaining the integrity of this study on students' experiences of transformative learning in the context of marginalised peoples (Braun and Clarke 2021)—in this case, Aboriginal peoples in Australia. Both researchers identify as non‐Aboriginal academics. Although we have extensive experience teaching and researching the experiences of Aboriginal peoples, we do not possess lived experience of Aboriginal cultures ourselves.

We recognise that this work is conducted within a system shaped by colonial histories that have long marginalised Aboriginal knowledge while privileging Western epistemologies. Our positionality therefore carries an inherent risk of misinterpreting or oversimplifying students' accounts. To mitigate this risk, we engaged in sustained reflexive practice, including vigilance towards our own assumptions, deep engagement with Aboriginal stories and relevant theoretical frameworks, and consistent application of cultural safety principles throughout all stages of the research process. We also discussed our potential biases concerning the strengths of the assessment and the Module itself, which were originally co‐written by one of the researchers and were taught by the other researcher in the semester the study was undertaken. To mitigate this, we ensured all findings, both positive and negative, were considered openly and objectively, including areas to further improve the delivery of the unit and assessment.

### 6.2 Interpretation of Student Transformative Learning Experiences

Through the analysis of reflective assessment data, the learning experiences of postgraduate nurse students undertaking and reflecting on the Ngany Kalyakal Kaatidjiny Boordawan Module have been described. More specifically, aspects of the transformative learning process have been identified, including the way students perceive this learning may influence their nursing practice.

These findings are now interpreted against their significance to nurse education learning and assessment activities, and the future cultural safety of the Australian nursing workforce. The discussion begins with the students' initial lack of cultural awareness. It then moves on to an examination of the students' transformative learning experiences and the role of reflective practice in the associated development of skills, perspectives and future plans for practice that align with the principles of culturally safe practice.

### 6.3 Cultural Awareness and Nurse Education

The importance of culture to health behaviours, experiences and outcomes and the central place of equity and empathy in nursing practice have been recognised for many decades (McGough et al. 2022). An early leader in transcultural nurse education, Leininger (1996), has described nursing as a transcultural phenomenon in which knowledge of patients' cultural values, beliefs and practices is crucial to effective holistic practice. In Australia, the ongoing lack of health equity experienced by Aboriginal peoples makes Aboriginal culture and health a prominent focus for undergraduate nursing education (ANMAC (2019); NMBA 2018).

The postgraduate nurses in our study described how they became aware of their own lack of Aboriginal cultural awareness during their engagement with the Module and reflections on this experience. Insufficient understanding of the impact of colonisation on Aboriginal health and wellbeing was particularly evident. This understanding is central to the prevention of culturally unsafe practice including incidents of racism (Allison et al. 2023).

With the exception of the migrant nurses in our study who may have undertaken their undergraduate nursing degree abroad, this lack of cultural awareness raises questions about the impact of Aboriginal culture and health content in Australian pre‐registration nursing degrees. As most of our participants were aged over 40, it may be that many of the Australian educated nurses had not benefitted from the mandated embedding of cultural content from 2009 (ANMAC 2019). The level of cultural study content in the preceding decades has been referred to as ‘minimal’ (Pinikahana et al. 2003).

A further barrier to the development of a culturally aware nursing workforce is the lack of evaluation of the inclusion of Aboriginal content in nursing undergraduate degree programmes (Laccos‐Barrett et al. 2022). Our findings offer new evidence that suggests this impact may be inadequate to reduce racism and enhance culturally aware and safe practice. The ongoing racism reported in nursing practice further indicates a lack of success of this initiative (Best et al. 2025).

The Australian approach to health education in nursing degrees also appears to contribute to the lack of culturally safe approaches to nursing. Most notably, nurses are predominantly educated through a Western biomedical health model (McGough et al. 2022), which ignores or minimises the impact of social determinants on health outcomes, including prejudice, politics, culture and structural factors. As such, this education leaves Australian nurses without the requisite skills to support Aboriginal communities and health outcomes (McGough et al. 2022).

### 6.4 Wider Influences on Nurses' Cultural Awareness

Lee and Greene (2003) argue that a student's understanding is influenced by factors outside the university, including upbringing, culture, schooling, religion and personal experiences. Our students reexamined a range of personal and family perspectives as well as societal norms as influences on their personal lack of cultural awareness. Students' use of terms such as ‘biassed’ and ‘naive’ to describe the influences of past norms and taken for granted family histories and their influence. This omission in schooling was identified in a few of the older students in our study.

The dearth of time in the school curriculum dedicated to Aboriginal culture and history including the impact of colonisation (Norman and Payne 2025) has been identified as a contributing factor to racist behaviours and perspectives among older Australians (aged 35 years and above). Through the unit assessment activity, students were able to actively reexamine the norms that shaped their previously unhelpful perspectives of Aboriginal culture and health, potentially for the first time.

### 6.5 The Importance of Meaningful Learning Experiences

This process of ascribing meaning to personal experiences can increase the significance of the experience to the learner (Savicki and Price 2021). In our study, participants described how they were able to make meaning of Aboriginal experiences through a process of identification with aspects of the Aboriginal stories presented in the Module. The process of personal identification with Aboriginal experiences of racism, prejudice and the impact of colonisation was most notable among the migrant students in the study. These students described their own experiences of racism as non‐white people in Australia and wrote about a growing sense of empathy with Aboriginal peoples as a result. Their empathy with other populations who had experienced British colonisation and resultant disempowerment and erosion of culture was evident. This and other examples of identification supported the development of empathy among participants. This increased capacity for empathy is a crucial attribute of culturally safe nursing, and a recognised benefit of reflective activities among nursing students (Yang and Zou 2025).

### 6.6 Critical Reflection Activities That Promote a Re‐Examination of Nursing Perspectives and Practice

McGough et al. explain ‘only when a nurse begins to unpack their own values and beliefs can they begin to understand the lens with which they view the world and consider a different outlook that recognises privilege and acknowledges the enduring impact of colonisation on the lives of Indigenous peoples today’ (2022, 37). The frame of reference through which adults perceive, experience and interact within the world described by Mezirow (2000), was challenged for and by the students' critical reexamination of personal and wider nursing perspectives and practices. The reexamination of the origins of unhelpful ideas around Aboriginal peoples provided a basis on which to challenge this frame of reference. Through this activity, students became more open to new, more inclusive perspectives on culture and difference. Students provided a range of examples where their previous lack of understanding of Aboriginal behaviours was resolved by their engagement with Module activities, including traditional ideas of death and family commitment.

### 6.7 Exploring Complex Concepts Through Critical Reflection Activities

Nurses today require flexible high order conceptual thinking skills to critically interpret and manage unfamiliar situations within an ever changing and increasingly challenging healthcare environment (Tsimane and Downing 2019). The application of reflective practice as a tool to support understanding of difficult and confronting concepts (Tsimane and Downing 2019) is pertinent in our study. The assessment activity enhanced the students' capacity to critically interpret concepts of equity, power and privilege, Western dominant ethnocentric ideas of health and the limitations of the biomedical model of health as a teaching model in nurse education.

Arguably a central pivotal concept in Aboriginal cultural safety practice is equity. CATSINaM explain that cultural safety is a philosophy of care provision according to unique need (equity) rather than the provision of the same care, regardless of need (equality) (2017).

In practice, the ideas of equity and equality can be confusing for nursing students as both are associated with fairness. Further, the social justice philosophy can be confronting to some who align with the idea of equality without an understanding of the higher needs of certain cultural and social groups due to unfair societal situations (Valderama‐Wallace and Apesoa‐Varano 2019).

### 6.8 Translating Transformative Learning Into Culturally Safe Practice

Hunt et al. (2015) describe how the process of transformative learning stimulates the development of self‐confidence and competence in new roles and relationships and ultimately brings about change in working environments. The transformative learning outcomes for our students included this increased confidence in working effectively and safely with Aboriginal peoples. Students also described an intention to engage in ongoing professional development activities, including training, collaboration and learning from and with Aboriginal colleagues and patients. Participants also discussed the importance of the explicit demonstration of respect for Aboriginal tradition in practice, including art and traditional ceremonies. This consideration of the students' transformative learning to practice intent is an important outcome of the process.

### 6.9 Planning and Introducing Reflection Activities With Care

While it is evident the reflective activities described in this project supported student growth in terms of cultural awareness and skills and perspectives associated with culturally safe practice, it also elicited discomfort for the students engaged in the process. The confronting nature of the Module content as well as the participants' self‐examination of past perspectives and practices intensified feelings of shame, guilt and distress. These concerns have been reported in other studies where non‐Aboriginal health professionals learn about the issues of racism, disadvantage and white privilege and their impact on Aboriginal lives (Norman and Payne 2025). While reflection relies on feelings of discomfort to prompt reexamination of perspectives, it can also cause individuals to shut down from processes that trigger these feelings, halting the learning process (Norman and Payne 2025). This finding prompted us to explore and embed support strategies in future Module delivery, such as safe space group discussion where feelings are shared, validated and normalised (Dunne et al. 2023).

### 6.10 Reflection Assessments and Artificial Intelligence

Although outside the immediate scope of this article, critical reflection assessments are recognised as less able to rely on artificial intelligence (AI). With the rise of reliance on artificial intelligence in academic writing, the Tertiary Education Quality and Standards Authority (TEQSA) has called for learning tasks that reveal student thinking processes, competencies and other learning skills and qualities (TEQSA 2023). The assessments described in this study provide these opportunities and as such are more difficult to simulate via AI than traditional assessments, such as essays and presentations on learning.

## 7 Limitations

The study reports on one university's work to promote culturally aware and safe nursing practice. Several limitations reduce the generalisability of these findings, including a small, self‐selected sample and a low response rate (33%) that introduces potential non‐response bias. Additionally, students may have engaged in performative writing because they were submitting these assessments for grading. Despite these constraints, interpreting the data through established theories of critical reflection and transformative learning enhances the relevance of these findings and their implications for nursing education across Australia. While individuals can become culturally safe through critical reflection activities described in this article and may develop the skills and intent to change their own practice, the development of a culturally safe service requires a systemic paradigm shift, with governance, leaders, funders and providers working together to challenge racism and prejudice as well as ignorance (McGough et al. 2022).

## 8 Conclusions

The critical assessment activity undertaken by the postgraduate nursing students engaged in an Aboriginal history and culture Module promoted transformative learning through a meaningful and powerful learning experience. Initial cultural awareness deficits were challenged and frames of reference were shifted to embrace knowledgeable, inclusive and respectful perspectives. Students developed a range of skills and attributes aligned with culturally safe practice.

## 9 Implications for Practice

The findings of this study have several implications for nursing education in preparing graduate nurses to practise in culturally aware and culturally safe ways.

First, nursing educators should not assume that students—whether undergraduate or postgraduate, Australian‐trained or internationally educated—have prior knowledge of Aboriginal culture, history or health needs. Consequently, all nursing curricula should include content on Aboriginal history, culture and ways of knowing, being and doing.

Second, the source and delivery of this content are critical. Nurse educators should collaborate with Aboriginal leaders and communities in the design and development of learning materials and educational activities. The inclusion of authentic Aboriginal voices and stories is particularly important for fostering meaningful student engagement and understanding.

Third, critical reflection activities embedded within assessments and learning experiences can support the transformative learning required for the development of cultural awareness. These activities provide opportunities for students to examine their assumptions, attitudes and biases, and to develop the skills and attributes necessary for culturally safe, non‐racist nursing practice.

Finally, educators should carefully consider how critical reflection activities are facilitated, particularly when addressing the sensitive and potentially confronting topic of Aboriginal history in Australia. Ongoing evaluation and refinement of these educational approaches are essential to ensure they effectively meet students' learning needs. Similarly, the broader impact of the Australian Nursing and Midwifery Accreditation Council (ANMAC) directive (2019), which requires the inclusion of Aboriginal cultural and historical content in undergraduate nursing programmes, should continue to be monitored and evaluated to inform future curriculum development.

## Author Contributions

Lesley Andrew: data curation, formal analysis, investigation, methodology, project administration, validation, visualisation, writing – original draft. Bev Ewens: conceptualization, formal analysis, investigation, review and editing.

## Funding

The authors have nothing to report.

## Conflicts of Interest

The authors declare no conflicts of interest.

## Data Availability

The data that support the findings of this study are available from the corresponding author upon reasonable request. The data are not publicly available due to privacy/ethical restrictions.

## Appendix 1

Brief Outline of Assessment Instructions:

Assessment one is a critical reflection on the Ngany Kalyakal Kaadidjiny Boordawan module.

Purpose of this assessment:

This assessment helps you to reflect on and critique your own values and attitudes. It supports your personal and professional development in terms of your interactions with Aboriginal and Torres Strait Islander Peoples as patients, clients and community. The assessment asks for an honest and open discussion of your reflection and learning.

Throughout your reflection discuss your learning with reference to the concepts introduced within the unit so far. These may include equity and equality, inter‐generational trauma, social determinants of health, etc. Explain how your learning from your reflection will influence your own nursing practice.

## Appendix 2 COREQ (COnsolidated Criteria for REporting Qualitative Research) Checklist

**Table:**

Topic	Item No.	Guide Questions/Description	Reported on Page No.
**Domain 1: Research team and reflexivity**			
*Personal characteristics*			
Interviewer/facilitator	1	Which author/s conducted the interview or focus group?	NA
Credentials	2	What were the researcher's credentials? e.g., PhD, MD	Title page
Occupation	3	What was their occupation at the time of the study?	8
Gender	4	Was the researcher male or female?	Female
Experience and training	5	What experience or training did the researcher have?	Title page
*Relationship with participants*			
Relationship established	6	Was a relationship established prior to study commencement?	8
Participant knowledge of the interviewer	7	What did the participants know about the researcher? e.g., personal goals, reasons for doing the research	8
Interviewer characteristics	8	What characteristics were reported about the inter viewer/facilitator? e.g., Bias, assumptions, reasons and interests in the research topic	19
**Domain 2: Study design**			
*Theoretical framework*			
Methodological orientation and Theory	9	What methodological orientation was stated to underpin the study? e.g., grounded theory, discourse analysis, ethnography, phenomenology, content analysis	8
*Participant selection*			
Sampling	10	How were participants selected? e.g., purposive, convenience, consecutive, snowball	8
Method of approach	11	How were participants approached? e.g., face‐to‐face, telephone, mail, email	8
Sample size	12	How many participants were in the study?	9
Non‐participation	13	How many people refused to participate or dropped out? Reasons?	9
*Setting*			
Setting of data collection	14	Where was the data collected? e.g., home, clinic, workplace	8
Presence of nonparticipants	15	Was anyone else present besides the participants and researchers?	8
Description of sample	16	What are the important characteristics of the sample? e.g., demographic data, date	9
*Data collection*			
Interview guide	17	Were questions, prompts, guides provided by the authors? Was it pilot tested?	NA
Repeat interviews	18	Were repeat interviews carried out? If yes, how many?	NA
Audio/visual recording	19	Did the research use audio or visual recording to collect the data?	NA
Field notes	20	Were field notes made during and/or after the interview or focus group?	NA
Duration	21	What was the duration of the interviews or focus group?	NA
Data saturation	22	Was data saturation discussed?	NA
Transcripts returned	23	Were transcripts returned to participants for comment and/or correction?	NA
**Domain 3: analysis and findings**			
*Data analysis*			
Number of data coders	24	How many data coders coded the data?	8
Description of the coding tree	25	Did authors provide a description of the coding tree?	No
Derivation of themes	26	Were themes identified in advance or derived from the data?	8
Software	27	What software, if applicable, was used to manage the data?	5
Participant checking	28	Did participants provide feedback on the findings?	No
*Reporting*			
Quotations presented	29	Were participant quotations presented to illustrate the themes/findings? Was each quotation identified? e.g., participant number	10–17
Data and findings consistent	30	Was there consistency between the data presented and the findings?	10–17
Clarity of major themes	31	Were major themes clearly presented in the findings?	9–17
Clarity of minor themes	32	Is there a description of diverse cases or discussion of minor themes?	10–17

*Note:* Developed from: Tong et al. (2007).
